# Copper – a novel stimulator of autophagy

**DOI:** 10.15698/cst2020.05.218

**Published:** 2020-04-24

**Authors:** Hans Zischka, Guido Kroemer

**Affiliations:** 1 Institute of Molecular Toxicology and Pharmacology, Helmholtz Center Munich, German Research Center for Environmental Health, Ingolstaedter Landstrasse 1, 85764 Neuherberg, Germany.; 2 Technical University Munich, School of Medicine, Institute of Toxicology and Environmental Hygiene, Biedersteiner Strasse 29, 80802 Munich, Germany.; 3 Centre de Recherche des Cordeliers, Equipe labellisée par la Ligue contre le cancer, Université de Paris, Sorbonne Université, Inserm U1138, Institut Universitaire de France, Paris, France.; 4 Metabolomics and Cell Biology Platforms, Institut Gustave Roussy, Villejuif, France.; 5 Pôle de Biologie, Hôpital Européen Georges Pompidou, AP-HP, Paris, France.; 6 Suzhou Institute for Systems Medicine, Chinese Academy of Medical Sciences, Suzhou, China.; 7 Karolinska Institute, Department of Women's and Children's Health, Karolinska University Hospital, Stockholm, Sweden.

**Keywords:** autophagy, Wilson disease, copper, cancer, ULK1, ULK2, MEK1

## Abstract

Toxic copper accumulation causes Wilson disease, but trace amounts of copper are required for cellular and organismal survival. In a recent paper Tsang *et al.* (Nat Cell Biol, doi: 10.1038/s41556-020-0481-4) demonstrate that copper binds with high affinity to a designated interaction site in the pro-autophagic kinases ULK1 and ULK2. Chelation of copper or genetic deletion of this copper-binding site inhibits autophagy and hence reduces the fitness of KRAS-induced cancers. These findings suggest that copper chelation might constitute a novel therapeutic intervention on autophagy-dependent malignancies.

In biomedicine, the transition metal copper has a Janus-faced dual role, as an essential but static cofactor of vital enzymes but also as a highly detrimental poison, if in excess. This vision is best exemplified by two deadly genetic diseases affecting copper concentrations in human tissues in opposed ways: Menkes disease, in which systemic copper uptake is deficient, and Wilson disease (WD), in which copper excretion from the body is blocked.

In WD, a steadily copper pile-up has long been thought to elicit omnipresent toxic oxidative stress, ultimately sealing cellular and organismal fate. While this may be true for the terminal phase of WD in which massive copper overload causes liver failure, an increasing body of evidence demonstrates that such uncontrolled Fenton chemistry reactions do not drive disease pathogenesis. Rather, it is increasingly recognized that copper directly interacts with target proteins, thus interfering with protein homeostasis and igniting cell death pathways [1], or with key enzymes involved in cellular bioenergetics, as shown for WD [2]. Of note, the copper pile-up in WD is paralleled by the activation of autophagy, a highly regulated cellular mechanism that destroys dysfunctional cytoplasmic components and allows their recycling, aiming at bioenergetic restoration [3] (**Figure 1**).

**Figure 1:**
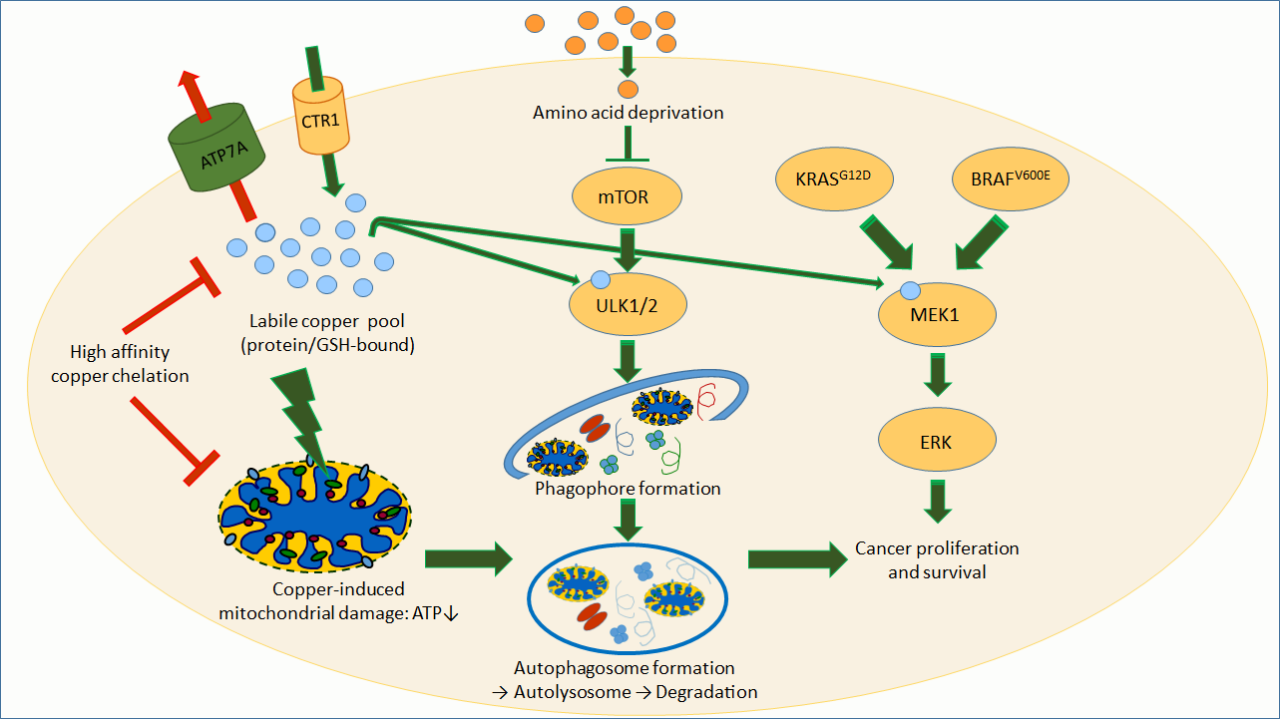
FIGURE 1: Copper-driven autophagy and its impact on Wilson disease and cancer. Cellular copper entry occurs via the transporter CTR1, and its efflux is driven by ATP7A (impaired in Menkes disease, or by ATP7B that is impaired in Wilson disease). High-affinity chelators, CTR1 depletion or ATP7A overexpression reduce the available intracellular copper-pool that otherwise may bind to autophagy activating kinases ULK1 and 2. Upon amino acid deprivation, mTOR kinase activity is reduced, thereby initiating the autophagic process in dependence of ULK1/2. In Wilson disease, excessive copper accumulates in mitochondria, causing bioenergetic deficits, thus providing a further pro-autophagic signal, and copper-burdened mitochondria are subjected to autophagic degradation. In certain types of cancer (e.g. KRAS^G12D^ lung adenocarcinomas), copper activates MAPK signaling via MEK1 besides ULK1/2 activation, thereby providing a dual signal that improves cancer cell survival and proliferation.

Recently, this view on a targeted copper action has been substantially strengthened by findings in certain cancers (e.g. BRAF^V600E^-positive melanomas). Thus, copper bound to the mitogen-activated protein kinase kinase 1 (MEK1) enhances its phosphorylation, thereby promoting tumorigenesis [4]. This MEK1 activation is due to copper binding to a histidine- and methionine-containing protein sequence of enormous copper affinity (with an apparent dissociation constant ~10^18^) [5]. Moreover, copper depletion with high affinity chelators turned out to be antineoplastic in such melanomas [6] (**Figure 1**). These findings strongly suggest copper binding to act as a novel post-transcriptional modification of proteins that play critical roles in cellular signaling.

In direct agreement with this vison, Tsang *et al.* found that two pro-autophagic kinases, Unc-51 like autophagy activating kinases 1 and 2 (ULK1, ULK2) possess MEK1-like copper-binding sequences [7]. *In vitro*, ULK1 and 2 do not bind iron or zinc, but copper, which dose-dependently increases their kinase activity. Conversely, ULK1 and 2 activities can be blocked by copper depletion with a high affinity copper chelator. At the cellular level, the authors showed that increasing the intracellular copper level by either expressing the high-affinity copper uptake transporter CTR1 (encoded by the SLC31A1 gene) or by depleting the copper export transporter ATP7A (also known as Menkes' protein, a copper-transporting P-type ATPase), increases ULK1/2 activity. Such copper-dependent ULK1/2 activation does stimulate autophagic flux, i.e. increased autophagic degradation activity, as indicated by enhanced lipidation of microtubule-associated proteins 1A/1B light chain 3B (LC3), as well as the abundance and ratio of autophagosomes and autolysomes. Moreover, upon autophagy activation by amino acid deprivation, intracellular copper levels increase, as determined by means of a copper-sensitive fluorophore. Conversely, the authors also found that copper deficiency decreases the formation of autophagy-initiating phagophores, which contain important ULK1/2 downstream targets (**Figure 1**. Finally, when expressing an ULK1 mutant that lacks the histidine-methionine copper binding sequence, Tsang *et al.* observed reduced ULK1 kinase activity as well as a significantly reduced autophagic flux in response to amino acid deprivation [7]. Altogether, these findings are compatible with the conclusion that copper binding to ULK1/2 is necessary and sufficient to increase autophagic flux.

What is the pathophysiological relevance of copper-initiated autophagy? In order to assess this issue, Tsang *et al.* studied KRAS-driven lung carcinomas that depend on efficient autophagy for tumor maintenance. Indeed, upon genetic ablation of the copper uptake transporter CTR1, a maneuver that lowers intracellular copper, autophagy is decreased in a mouse model of KRAS-driven lung cancer, paralleled by a sizeable reduction of tumorigenesis. Moreover, upon blocking copper binding to ULK1 (by replacing endogenous ULK1 by a mutant form lacking the copper binding site), tumor growth kinetics are reduced, resulting in significantly lower tumor weight in a xenograft model. These results document the role of autophagy in sustaining the growth of KRAS-driven tumors. Most importantly, however, the study by Tsang *et al.* highlights the fundamental role of copper in tumorigenesis. In this scenario, copper-binding, activated MEK1 emits a permanent growth signal. However, blocking such a Cu-stimulated MAPK signaling (e.g. by specific inhibitors) is not sufficient for causing growth arrest and cell death, as cytoprotective Cu-mediated autophagy is activated as well. The resulting copper dependence of vital cancer pathways may provide a teleological explanation for the two- to fourfold elevated copper content in certain tumor types, that was reported almost one-hundred years ago [8].

Taken together, these studies profoundly challenge the concept of oxidative stress as the mere or prime cellular mediator of intracellular copper action, but rather highlight much subtler actions of this transition metal. The resulting new vision blurs the black-and-white picture of antinomic beneficial versus detrimental copper effects, as an increased autophagic flux may have a variety of consequences, depending on the circumstances [9]. In the context of specific malignancies, unsustainable growth or tumor escape from chemotherapy may result from increased copper concentrations. Thus, as suggested by Tsang *et al.*, the repurposing of copper chelators employed against copper overload may be of outstanding value, as some of these chelators have been clinically used against WD for several decades. Chelators with very high affinity may directly interfere with copper-driven signaling events, whereas medium affinity chelators may interfere with systemic copper uptake. In vulnerable cancers, copper chelators may be advantageously combined with cell death initiating compounds [10]. Given the low toxicity of such copper chelators, their broad availability, clinical studies evaluating their therapeutic utility against cancer are urgently awaited.
